# Renal Subcapsular Hematoma Due to Reperfusion Injury Following Renal Angioplasty in Fibromuscular Dysplasia: A Dilemma in Diagnosis and Management

**DOI:** 10.7759/cureus.23350

**Published:** 2022-03-21

**Authors:** Vanlalmalsawmdawngliana Fanai, Animesh Mishra, Tony Ete, Amit Malviya, Arun Kumar

**Affiliations:** 1 Cardiology, North Eastern Indira Gandhi Regional Institute of Health and Medical Sciences, Shillong, IND

**Keywords:** ptra, reperfusion injury, resistant hypertension, renal subcapsular hematoma, fibromuscular dysplasia

## Abstract

Fibromuscular dysplasia (FMD) is a potentially treatable cause of renovascular hypertension and it typically affects young females. FMD usually involves distal two-thirds of the renal artery and percutaneous transluminal renal angioplasty (PTRA) is the treatment of choice for FMD with resistant hypertension. PTRA is a safe procedure with minimal complications. However, renal subcapsular hematoma due to reperfusion injury is a rare complication following PTRA. A 32-year-old male presented with resistant hypertension. Arteriography of renal arteries showed >90% stenosis of ostial-proximal left renal artery with a string of beads appearance. PTRA was performed with the deployment of a 4 x 10 mm balloon-expandable stent in the stenotic segment of the left renal artery. However, computed tomography of the abdomen revealed massive left perinephric subcapsular hematoma without peritoneal collection. As the patient was hemodynamically stable, no invasive intervention was done, and discharged without requiring any anti-hypertensive medication. Putative reperfusion injury may provoke bleeding complications after renal angioplasty in a case of long-standing renal artery stenosis and can be managed conservatively with close surveillance in certain cases.

## Introduction

Fibromuscular dysplasia (FMD) is non-inflammatory and non-atherosclerotic vasculopathy that affects medium and large arteries. Renal artery FMD is the second most common cause of renovascular hypertension, especially among women and the young. In contrast, atherosclerosis is the most common cause of renovascular hypertension and mostly affects the elderly with other cardiovascular risk factors. Atherosclerosis typically involves the ostial-proximal segment of the renal artery and FMD usually affects its distal two-thirds. Identification of the renal artery FMD is crucial, as it is successfully treated with revascularization therapy, especially in patient with resistant hypertension. Percutaneous transluminal renal angioplasty (PTRA) is a safe procedure with minimal complications, the most common being hematoma at the vascular access site. Renal subcapsular hematoma due to reperfusion injury is a rare complication following PTRA and is rarely described in the literature. Here, we report a case of a renal subcapsular hematoma, a rare complication of PTRA due to reperfusion injury in a young hypertensive male with FMD involving an ostial-proximal segment of the left renal artery.

## Case presentation

A 32-year-old male, diagnosed case of young systemic hypertension presented to us with resistant hypertension. He had a history of intermittent pulsatile headache, dyspnea on exertion (NYHA II), and recurrent epistaxis for the past eight months. Besides systemic hypertension, no other cardiovascular risk factor was found. At the time of admission, he was afebrile with resting blood pressure in the right upper limb was 190/120 mmHg and 186/120 in the left upper limb, pulse rate was 62/min (regular). On systemic examination, an abdominal bruit was heard over the left lumbar region, and the rest of the clinical examinations were unremarkable.

**Figure 1:**
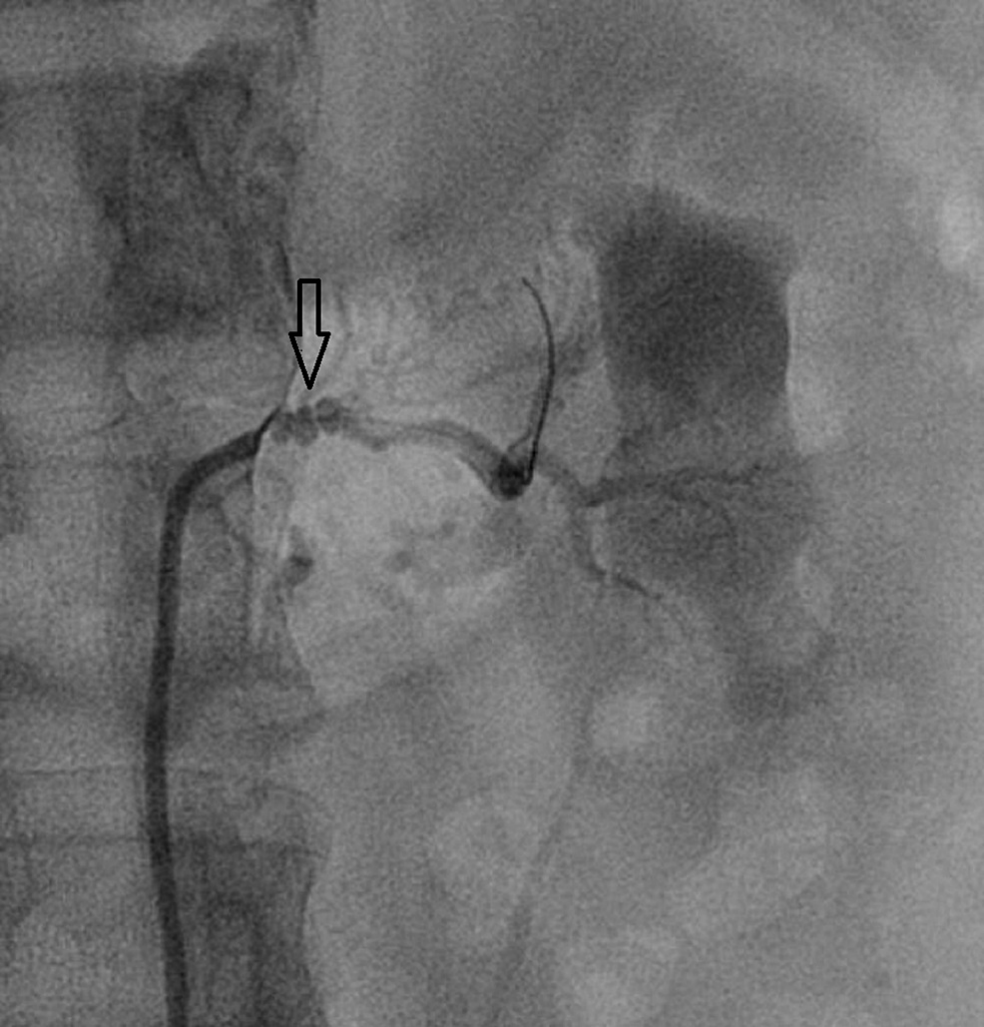
Renal angiography showing >90% stenosis of an ostial-proximal segment of the left renal artery with a string of beads appearance.

On further evaluation, abdominal Doppler ultrasound revealed the normal size and echotexture of the right kidney; however, the left kidney was contracted (7.8x5.6cm) with elevated flow velocity in the left renal artery (peak systolic velocities 378 cm/sec). Arteriography of renal arteries was performed via right femoral artery using 5F JR diagnostic catheter, which confirmed >90% stenosis of ostial-proximal left renal artery with a string of beads appearance (Figure 1).

Baseline laboratory data were within normal range including kidney function test, erythrocyte sedimentation rate, and C-reactive protein (CRP).

Despite administration of multiple appropriate antihypertensive drugs (carvedilol 25mg BD, prazosin 10mg OD, amlodipine 10mg OD, chlorthalidone 50mg OD), his blood pressure remained uncontrolled. Ramipril 10mg OD was added as the baseline kidney function was normal and the lesion was confined to left renal artery only. As the blood pressure was persistently high PTRA was performed to control resistant hypertension.

The PTRA was performed through the right femoral artery using 6F RDC (renal double curve) guiding catheter and Fielder guidewire, a 4 x 10 mm balloon expandable stent (Herculink Elite Abbott) was deployed at the stenotic area of the left renal artery. As the procedure was uneventful and angiogram showed well perfused renal artery without complications, intravascular imaging was not performed. The systemic blood pressure improved instantaneously following stent placement. However, soon after completion of the procedure the patient developed pain over the left flank. Repeat angiography was done immediately and possible complications like perforation, dissection and stent closure were ruled out.

**Figure 2:**
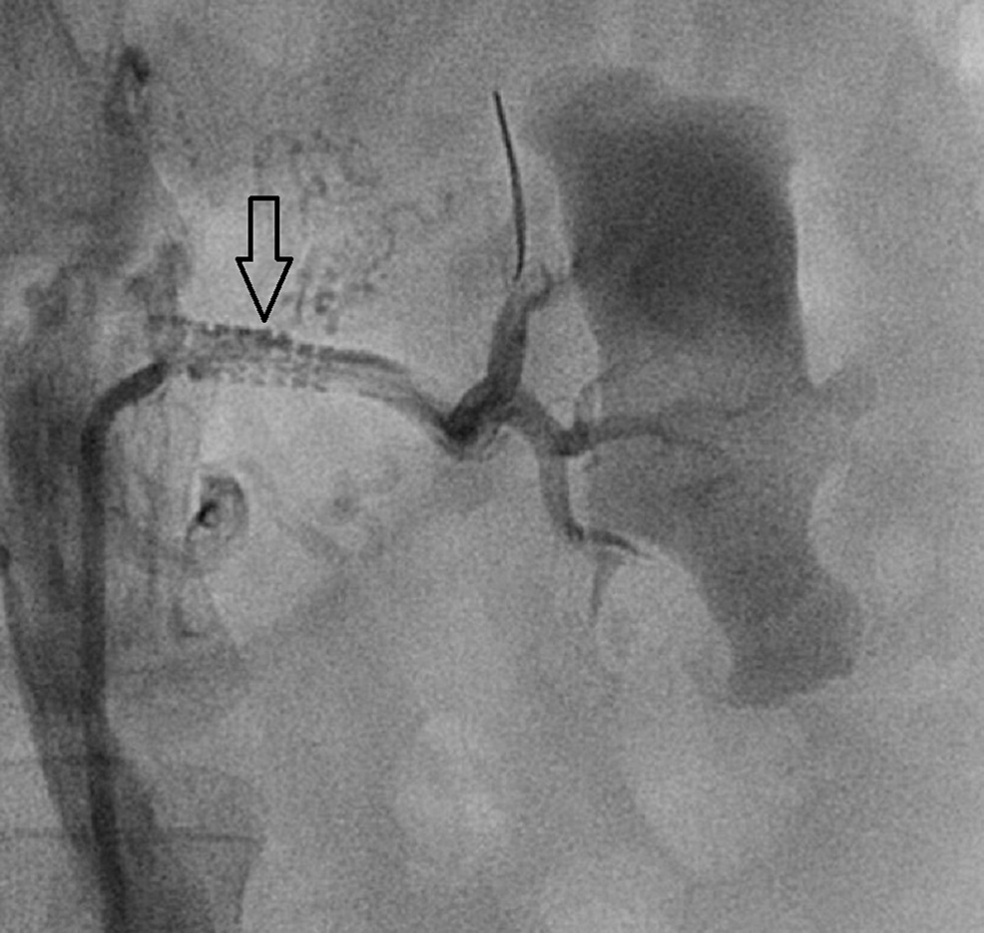
Angiogram after percutaneous transluminal renal angioplasty showing well deployed stent without evidence of perforation, dissection, or stent closure.

After he was shifted to intensive coronary care unit (ICCU), the pain was still persisting and was associated with tachycardia (approximately 120-140/min) and excessive perspiration. An urgent bedside ultrasound was performed which revealed small isoechoic area in the left perinephric region, suggestive of perinephric fluid collection. Due to significant fall in hemoglobin (Hb) level on postoperative day 1 (9.5 g/dL from 14 g/dL), an urgent contrast enhanced computed tomography (CECT) of abdomen was advised. CECT revealed massive left perinephric subcapsular hematoma (7.8 x 4.7 x15.3 cm) without peritoneal collection (Figure 2).

**Figure 3:**
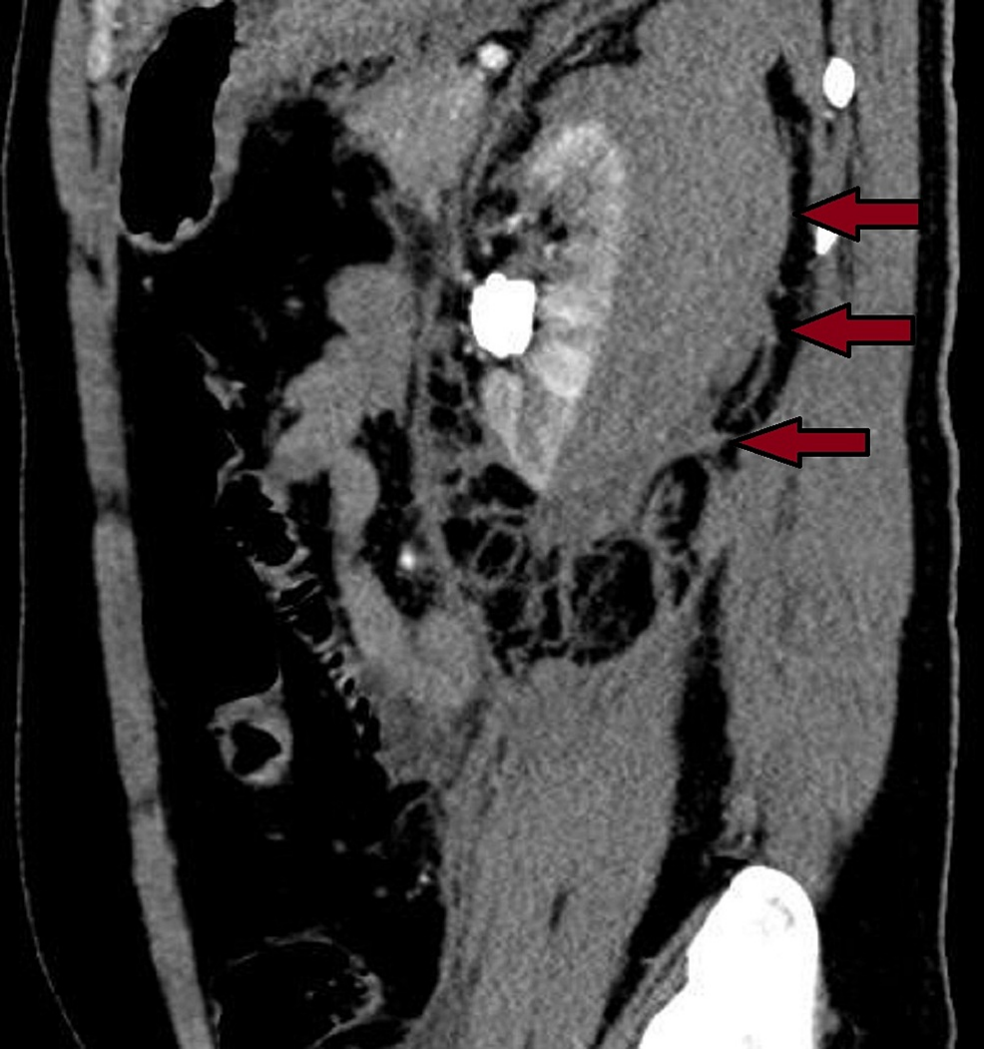
Computed tomography (CECT) of the abdomen showing massive left perinephric subcapsular hematoma (7.8 x 4.7 x 15.3 cm) without peritoneal collection.

Since the patient was otherwise hemodynamically stable, no further intervention was done and was treated conservatively along with close observation. During hospital stay he developed hyperbilirubinemia due to extravascular hemolysis (total bilirubin - 9.5 mg/dL, conjugated bilirubin - 4.9 mg/dL, ALT-44 U/L, AST-52 U/L, ALP-100 U/L, and LDH-620 U/L) and it resolved spontaneously over a period of two weeks. His Hb subsequently improved after three units of blood transfusion. Repeat CT scan on postoperative day 10 showed significant reduction of subcapsular hematoma (Figure 3).

Two weeks later, the general condition of the patient improved and he was discharged without needing any antihypertensive drug.

## Discussion

Renovascular hypertension is the most common identifiable and treatable cause of secondary hypertension. Though the prevalence is much higher in patients with acute and severe or refractory hypertension, it accounts for less than 1% of cases of mild to moderate elevations in blood pressure [1]. FMD is a non-inflammatory, non-atherosclerotic vascular disorder that affects medium and large arteries. It is the second most common cause of renal artery stenosis (RAS) next to atherosclerosis and accounted for 10% of the cases of RAS [2]. Beside stenosis, FMD is complicated by an aneurysm, dissection, and tortuosity of the affected arteries. FMD of renal artery mostly affect young female (female to male ratio 9:1) and typically involves middle or distal renal arteries, whereas atherosclerosis usually involves the ostium and proximal segment of the renal artery [3]. Therefore, the peculiar feature of the present case is FMD involving the ostial-proximal segment of the renal artery in a young male patient, presenting with resistant hypertension.

The etiology of FMD remains elusive and genetics may play an important role. The most common clinical manifestation of FMD involving renal artery is systemic hypertension. Clinical clues to suspect FMD has been mentioned in Table 1.

**Table 1:** Clinical signs of renal artery fibromuscular dysplasia

Hypertensive patients <30 years of age, especially in women
Accelerated, malignant, or grade 3 (>180/110 mmHg) hypertension
Drug-resistant hypertension (blood pressure target not achieved despite 3-drug therapy at optimal doses including a diuretic)
Unilateral small kidney without a causative urological abnormality
Abdominal bruit in the absence of atherosclerotic disease or risk factors for atherosclerosis
Suspected renal artery dissection/infarction
Presence of fibromuscular dysplasia in at least 1 other vascular territory

The mechanism of hypertension in FMD is secondary to activation of the renin-angiotensin-aldosterone system (RAAS), triggered by renal ischemia [4].

At present, FMD is virtually always diagnosed radiographically as histology is not always available. There are two angiographic subtypes of FMD: Multifocal FMD has the angiographic appearance of a “string of beads and is the most common type.” Pathologically it corresponds to medial fibroplasia. Focal FMD is less common and has the angiographic appearance of a “circumferential or tubular stenosis” and corresponds pathologically to intimal fibroplasia [5].

Treatment options for patients with renal artery FMD include medical therapy or revascularization (PTRA/surgery). As the underlying pathogenesis of hypertension is due to activation of the RAAS, the initial drug class of choice is an angiotensin-converting enzyme inhibitor (ACEi) or angiotensin receptor blocker (ARB) [5]. But ACEi and ARBs are notorious for deteriorating glomerular filtration rate (GFR), especially if the stenotic lesions are severe and occur bilaterally or in a solitary kidney. The primary target of revascularization in FMD is adequate to control blood pressure. Therefore, the risks of revascularization may outweigh the benefits in a patient with relatively well-controlled hypertension. Since there are no randomized trials comparing revascularization with medical therapy alone in patients with renal FMD, Olin et al. recommended revascularization under the following conditions: resistant hypertension, hypertension of short duration, severe stenosis (especially in the pediatric population), renal artery dissection (stenting is generally the procedure of choice), renal artery aneurysm and branch renal artery disease [5].

Till date, there are no comparative trials, but PTRA is considered safe and effective if performed by experienced hands. The reported technical (angiography) success rates for a PTRA range from 83% to nearly 100% [6,7]. Thus, it is suggested that most patients with FMD who are selected for renal revascularization should undergo PTRA rather than surgery. However, surgery is preferred if PTRA fails or if the arterial anatomy is not amenable to PTRA, as in patients with small renal arteries (<4 mm), with branch renal artery disease, or with extensive intimal fibroplasia [5].

Complications after PTRA are usually minor, with hematoma being the most common and mostly related to vascular access sites [8]. Major complications including hemorrhage, dissection, stent migration, vessel thrombosis, and emboli, are seen in up to 6.3% of patients with a mortality rate of 0.9% [9]. Though there were few related cases reported in the literature, the renal subcapsular hematoma which happened to this patient is a rare complication after PTRA [10,11]. Similar to our case, Hieronimus et al. also reported a renal subcapsular hematoma after stent angioplasty in a renal artery FMD. But unlike our case, the bleeding complication was successfully managed by endovascular coil embolization [12].

In the absence of direct injury to the renal artery during angioplasty such as perforation and dissection, the exact mechanism of renal subcapsular hematoma after PTRA is uncertain. One of the proposed mechanisms is reperfusion injury, as seen in cerebral hyperperfusion syndrome after endovascular treatment of high-grade carotid stenosis. According to this hypothesis, when there is a state of chronic low blood circulation in the brain, there is a compensatory dilatation of cerebral distal vessels to maintain adequate cerebral blood flow. In response to a sudden increase in blood pressure after the intervention, these chronically dilated small vessels lose their capacity to auto-regulate vascular resistance and result in a sudden increase in cerebral blood flow. Analogous to this mechanism, an excessive increase in renal blood flow soon after PTRA might rupture small distal renal vessels leading to the formation of renal subcapsular hematoma. In this case, as perforation and dissection were ruled out immediately by urgently repeated angiography (Figure 2), reperfusion injury was the most probable cause of renal subcapsular hematoma.

The management of renal subcapsular hematoma relies upon the clinical condition of the patient and the availability of a treatment facility. Similar to our case, Kang et al. managed their case conservatively without any intervention; while Hieronimus et al. successfully managed it by endovascular embolization [10,12].

## Conclusions

In any case of young hypertension, the treating physician must be aware of FMD as a possible cause since it is potentially treatable with intervention. This case suggested that reperfusion injury may provoke bleeding as a complication of renal angioplasty in a case of severe long-standing renal artery stenosis.

## References

[1] Textor SC, Lerman L (2010). Renovascular hypertension and ischemic nephropathy. Am J Hypertens.

[2] Olin JW, Froehlich J, Gu X (2012). The United States Registry for fibromuscular dysplasia: results in the first 447 patients. Circulation.

[3] Safian RD, Textor SC (2001). Renal-artery stenosis. N Engl J Med.

[4] Gornik HL, Persu A, Adlam D (2019). First international consensus on the diagnosis and management of fibromuscular dysplasia. J Hypertens.

[5] Olin JW, Gornik HL, Bacharach JM (2014). Fibromuscular dysplasia: state of the science and critical unanswered questions: a scientific statement from the American Heart Association. Circulation.

[6] Kløw NE, Paulsen D, Vatne K, Rokstad B, Lien B, Fauchald P (1998). Percutaneous transluminal renal artery angioplasty using the coaxial technique. Ten years of experience from 591 procedures in 419 patients. Acta Radiol.

[7] Birrer M, Do DD, Mahler F, Triller J, Baumgartner I (2002). Treatment of renal artery fibromuscular dysplasia with balloon angioplasty: a prospective follow-up study. Eur J Vasc Endovasc Surg.

[8] Dubel GJ, Murphy TP (2008). The role of percutaneous revascularization for renal artery stenosis. Vasc Med.

[9] Trinquart L, Mounier-Vehier C, Sapoval M, Gagnon N, Plouin PF (2010). Efficacy of revascularization for renal artery stenosis caused by fibromuscular dysplasia: a systematic review and meta-analysis. Hypertension.

[10] Kang KP, Lee S, Kim W, Han YM, Kang SK, Park SK (2007). Renal subcapsular hematoma: a consequence of reperfusion injury of long standing renal artery stenosis. Electrolyte Blood Press.

[11] Axelrod DJ, Freeman H, Pukin L, Guller J, Mitty HA (2004). Guide wire perforation leading to fatal perirenal hemorrhage from transcortical collaterals after renal artery stent placement. J Vasc Interv Radiol.

[12] Abou-Chebl A, Yadav JS, Reginelli JP, Bajzer C, Bhatt D, Krieger DW (2004). Intracranial hemorrhage and hyperperfusion syndrome following carotid artery stenting: risk factors, prevention, and treatment. J Am Coll Cardiol.

